# Supplementary material for: End Sequence Analysis Toolkit (ESAT) expands the extractable information from single-cell RNA-seq data
Source: Genome Res. 2016 Oct;26(10):1397–410. doi: 10.1101/gr.207902.116 (PMC5052061; doi:10.1101/gr.207902.116)
Supplement: Supplemental Material [file supp_gr.207902.116_Supp_Materials.tar.gz › ESAT-esat_v0.1/doc/html/classbroad_1_1core_1_1siphy_1_1_tree_scaler_i_o.html]

ESAT: broad.core.siphy.TreeScalerIO Class Reference


ESAT


- Main Page
- Related Pages
- Packages
- Classes
- Files

- Class List
- Class Index
- Class Hierarchy
- Class Members

All Classes Namespaces Files Functions Variables Enumerator Pages

- broad
- core
- siphy
- TreeScalerIO

Classes |
Public Member Functions |
List of all members

broad.core.siphy.TreeScalerIO Class Reference

Collaboration diagram for broad.core.siphy.TreeScalerIO:

[legend]

|  |  |
| --- | --- |
| Classes | |
| class | **ScaledWindow** |
|  | |

|  |  |
| --- | --- |
| Public Member Functions | |
|  | TreeScalerIO (int windowSize) |
|  | |
| void | loadIntoDistribution (File source, EmpiricalDistribution ed, boolean shortFormat) throws IOException |
|  | |
|  | TreeScalerIO () |
|  | |
| void | parse (File source, int shift, String chr) throws IOException |
|  | |
| void | load (InputStream is, int shift, String chr) throws IOException |
|  | |
| void | load (File source, int shift, String chr) throws IOException |
|  | |
| void | load (InputStream is) throws IOException |
|  | |
| void | loadIntegrated (File source, double minPVal) throws IOException |
|  | |
| void | loadOldIntegrated (File source, int shift, String chr, double minPVal) throws IOException |
|  | |
| void | addListener (ScaledWindowParserListener listener) |
|  | |
| void | loadAtMostRandom (File source, String chr, int maxNeutralOmegas) throws IOException |
|  | |
| void | stitch (int window, int skip) |
|  | |
| void | writeFull (BufferedWriter bw, List< ScaledWindow > windows) throws IOException |
|  | |
| void | writeFull (BufferedWriter bw) throws IOException |
|  | |
| void | writeFullAsGenomeGraphUsingLogPval (BufferedWriter bw) throws IOException |
|  | |
| void | addWindow (ScaledWindow sw) |
|  | |
| List< ScaledWindow > | getScaledWindows () |
|  | |
| void | clear () |
|  | |
| void | setNumberFormatter (DecimalFormat formatter) |
|  | |
| int | getEstimationWindow () |
|  | |
| void | setEstimationWindow (int estimationWindow) |
|  | |
| int | getTotalInSources () |
|  | |
| void | permute () |
|  | |

## Constructor & Destructor Documentation

|  |  |  |  |  |  |
| --- | --- | --- | --- | --- | --- |
| broad.core.siphy.TreeScalerIO.TreeScalerIO | ( | int | *windowSize* | ) |  |

|  |  |  |  |  |
| --- | --- | --- | --- | --- |
| broad.core.siphy.TreeScalerIO.TreeScalerIO | ( |  | ) |  |

## Member Function Documentation

|  |  |  |  |  |  |
| --- | --- | --- | --- | --- | --- |
| void broad.core.siphy.TreeScalerIO.addListener | ( | ScaledWindowParserListener | *listener* | ) |  |

|  |  |  |  |  |  |
| --- | --- | --- | --- | --- | --- |
| void broad.core.siphy.TreeScalerIO.addWindow | ( | ScaledWindow | *sw* | ) |  |

|  |  |  |  |  |
| --- | --- | --- | --- | --- |
| void broad.core.siphy.TreeScalerIO.clear | ( |  | ) |  |

|  |  |  |  |  |
| --- | --- | --- | --- | --- |
| int broad.core.siphy.TreeScalerIO.getEstimationWindow | ( |  | ) |  |

|  |  |  |  |  |
| --- | --- | --- | --- | --- |
| List<ScaledWindow> broad.core.siphy.TreeScalerIO.getScaledWindows | ( |  | ) |  |

Here is the caller graph for this function:

|  |  |  |  |  |
| --- | --- | --- | --- | --- |
| int broad.core.siphy.TreeScalerIO.getTotalInSources | ( |  | ) |  |

|  |  |  |  |
| --- | --- | --- | --- |
| void broad.core.siphy.TreeScalerIO.load | ( | InputStream | *is*, |
|  |  | int | *shift*, |
|  |  | String | *chr* |
|  | ) |  | throws IOException |

Here is the caller graph for this function:

|  |  |  |  |
| --- | --- | --- | --- |
| void broad.core.siphy.TreeScalerIO.load | ( | File | *source*, |
|  |  | int | *shift*, |
|  |  | String | *chr* |
|  | ) |  | throws IOException |

Here is the call graph for this function:

|  |  |  |  |  |  |
| --- | --- | --- | --- | --- | --- |
| void broad.core.siphy.TreeScalerIO.load | ( | InputStream | *is* | ) | throws IOException |

Here is the call graph for this function:

|  |  |  |  |
| --- | --- | --- | --- |
| void broad.core.siphy.TreeScalerIO.loadAtMostRandom | ( | File | *source*, |
|  |  | String | *chr*, |
|  |  | int | *maxNeutralOmegas* |
|  | ) |  | throws IOException |

|  |  |  |  |
| --- | --- | --- | --- |
| void broad.core.siphy.TreeScalerIO.loadIntegrated | ( | File | *source*, |
|  |  | double | *minPVal* |
|  | ) |  | throws IOException |

|  |  |  |  |
| --- | --- | --- | --- |
| void broad.core.siphy.TreeScalerIO.loadIntoDistribution | ( | File | *source*, |
|  |  | EmpiricalDistribution | *ed*, |
|  |  | boolean | *shortFormat* |
|  | ) |  | throws IOException |

|  |  |  |  |
| --- | --- | --- | --- |
| void broad.core.siphy.TreeScalerIO.loadOldIntegrated | ( | File | *source*, |
|  |  | int | *shift*, |
|  |  | String | *chr*, |
|  |  | double | *minPVal* |
|  | ) |  | throws IOException |

|  |  |  |  |
| --- | --- | --- | --- |
| void broad.core.siphy.TreeScalerIO.parse | ( | File | *source*, |
|  |  | int | *shift*, |
|  |  | String | *chr* |
|  | ) |  | throws IOException |

|  |  |  |  |  |
| --- | --- | --- | --- | --- |
| void broad.core.siphy.TreeScalerIO.permute | ( |  | ) |  |

|  |  |  |  |  |  |
| --- | --- | --- | --- | --- | --- |
| void broad.core.siphy.TreeScalerIO.setEstimationWindow | ( | int | *estimationWindow* | ) |  |

|  |  |  |  |  |  |
| --- | --- | --- | --- | --- | --- |
| void broad.core.siphy.TreeScalerIO.setNumberFormatter | ( | DecimalFormat | *formatter* | ) |  |

|  |  |  |  |
| --- | --- | --- | --- |
| void broad.core.siphy.TreeScalerIO.stitch | ( | int | *window*, |
|  |  | int | *skip* |
|  | ) |  |  |

|  |  |  |  |
| --- | --- | --- | --- |
| void broad.core.siphy.TreeScalerIO.writeFull | ( | BufferedWriter | *bw*, |
|  |  | List< ScaledWindow > | *windows* |
|  | ) |  | throws IOException |

Here is the caller graph for this function:

|  |  |  |  |  |  |
| --- | --- | --- | --- | --- | --- |
| void broad.core.siphy.TreeScalerIO.writeFull | ( | BufferedWriter | *bw* | ) | throws IOException |

Here is the call graph for this function:

|  |  |  |  |  |  |
| --- | --- | --- | --- | --- | --- |
| void broad.core.siphy.TreeScalerIO.writeFullAsGenomeGraphUsingLogPval | ( | BufferedWriter | *bw* | ) | throws IOException |

---

The documentation for this class was generated from the following file:

- java/broad/core/siphy/TreeScalerIO.java


---

Generated on Fri Aug 15 2014 15:10:57 for ESAT by  

 1.8.7
